# Reflections on Public Health in South Africa, 1993–2002

**DOI:** 10.3402/gha.v6i0.20094

**Published:** 2013-01-24

**Authors:** William Pick

News of the completion of the new Wits School of Public Health (WSPH) building reminded me of the dream that so many of us had when the School was founded just over a decade ago. At the time I wrote: ‘The year 2001 will be remembered as the year in which the Wits School of Public Health was established. This event marks a defining moment in the development of Public Health at Wits. The establishment of the school has over a short period of a few months brought a new dimension to the way in which we function. No longer can Public Health be regarded as the Cinderella of health sciences at Wits … the School has demonstrated its academic worthiness.’ The new building serves to reinforce this notion. The establishment of the School did not happen without opposition from within the Faculty of Health Sciences and the university. Similarly, finding a building suitable for the rapidly expanding School of Public Health did not happen without a struggle. Initial efforts led to the university architects drawing up plans for a multi-story School of Public Health building near the entrance of the Medical School. However, the university did not see its way clear to supporting such an undertaking and the plans were shelved. Efforts to acquire premises on Oxford Road, which members of staff inspected and liked, were similarly not supported by the university. It is for this reason, amongst others, that it is such a tremendous privilege to see the new building finally a reality – indeed a dream come true.

## Public Health: 1993–2002

Public Health at Wits, as elsewhere, was not the stuff that medical faculties were made of in the 1990s. Two factors influenced the trajectory of the academic discipline of Public Health significantly. The first was the dramatic political change in South Africa which led to the establishment of a new democratic order with human rights, including the right to health and health care, enshrined in the country's constitution. The School played a significant role in shaping national health policy in South Africa. In 1994, two of the nine ministerial committees were chaired by staff in the School, and many other staff members served on a range of committees and advisory bodies that ultimately influenced government thinking and health policies in the democratic era. The second major event was the HIV and AIDS disaster, which had a devastating effect on mortality and morbidity in South Africa and brought enormous challenges to its health system. These external factors had an impact on the development of Public Health in significant ways. The new government had a large constituency to whose needs it had to respond to. As a result, it looked to Public Health to address the dire health problems faced by a large majority of South Africans, while the HIV epidemic exposed the worst failings of the South African Public Health system.

Notwithstanding these imperatives, material support for academic Public Health was not forthcoming. It took a lot of nagging and some strong words to eventually gain seven additional academic posts from the Gauteng Health Department in 2002, bearing in mind that the WSPH only had about nine provincial Health Department funded posts at the time, some of which were ‘frozen’ during the implementation of financial austerity measures as part of the government's Growth Equity and Redistribution (GEAR) policy in the late 1990s.

The emergence of Public Health at Wits also benefited from the readmission of South Africa to the world community, leading to exposure to many academics prominent in international Public Health. The school benefited from a regular stream of international visitors from other parts of Africa, Latin America, Asia, the United States of America and the United Kingdom. In South Africa, the 1990s saw several initiatives to start regional schools of Public Health. Wits University was part of the five-university initiative, called the Transvaal School of Public Health (later named the Thusano School of Public Health or TSPH), which aimed to strengthen the discipline of Public Health in the northern regions of South Africa. All in the Faculty did not support the TSPH as some felt that trans-university courses and programmes would compromise Wits’ academic standards. These barriers had to be overcome and eventually an agreement at the level of the university Vice-Chancellors formalised the TSPH.

Following the World Development Report 1993, which argued the economic case for investing in health, there was a growing realisation that the global burden of diseases like malaria, HIV & AIDS and tuberculosis had a negative impact on the world economy. As a result, the world's rich nations established the Global Fund that supported the response to these three killer diseases. This gave added impetus to a worldwide resurgence of Public Health and provided much needed resources for Public Health research in South Africa.

In keeping with its commitment to strengthen South Africa's Public Health capacity, the WSPH initiated a Master of Public Health (MPH) programme and an MSc in Epidemiology in addition to the existing diploma courses, despite the enormous strain put on the limited teaching resources. This resulted in student numbers growing exponentially.

Meanwhile, the rural health initiatives started by John Gear, flourished. The Wits Rural Facility underwent a new incarnation; the Health Services (later Systems) Development Unit thrived, and spawned the Agincourt Project, which has since become one of the leading demographic and health surveillance sites in the world. Agincourt, in turn, gave birth to the Rural AIDS and Development Action Research programme (RADAR).

The Centre for Health Policy (CHP) excelled in the new post-apartheid environment and provided policy support for many government initiatives. It grew in size and the Women's Health Project, which it housed, became a leader in the struggle for gender equality in health care. This went on to have an enormous influence in shaping South Africa's new abortion legislation.

Urban Health was not to be left out as the School, as part of the WHO Collaborating Centre for Urban Health, continued to work in inner city areas such as Hillbrow and the Alexandra Township to the north of Johannesburg city centre. The emergence of Urban Health as a major field of endeavour internationally, led to the publication of the Urban Health Bulletin, the Healthy Cities movement and an increasing international collaboration of the School with partners in Europe, Africa, Latin America and South East Asia.

The novel addition of the Division of Oral Public Health enriched the academic endeavour of the WSPH, bringing much needed attention to the field of health promotion.

As I reflect on the Wits School in the 1990s, I am struck by the confluence of events, internationally and nationally, which provided a window of opportunity for Public Health to play a significant role in the improvement of the human condition. I have no doubt that the new School of Public Health building will stand as a beacon of hope for the improvement of population health, especially for the marginalised, dispossessed, oppressed and disempowered, in South Africa and beyond.

